# Synergistic enrichment and catalytic sensing platform based on ZIF-8-NH₂/dynamic Schiff base hydrogel for ultrasensitive detection of hydroquinone

**DOI:** 10.3389/fnut.2026.1772599

**Published:** 2026-04-20

**Authors:** Yang Chong, Fengsong Nie, Xiang Huang, Lingli Wang, Lingling Zhu, Shu Zhang, Junjun Yu, Yang Wang

**Affiliations:** 1 Department of Clinical Nutrition, The Affiliated Hospital of Yangzhou University, Yangzhou University, Yangzhou, Jiangsu, China; 2 School of Chemistry and Chemical Engineering, Yangzhou University, Yangzhou, China; 3 The First People's Hospital of Guannan County, Lianyungang, Jiangsu, China

**Keywords:** electrochemical sensing, food, hydrogel, hydroquinone, MOF, synergistic effect

## Abstract

**Introduction:**

The development of sensing interfaces that can simultaneously achieve efficient enrichment of targets and enhanced electron conduction is the key to improving the performance of phenolics detection in food

**Methods:**

In this paper, a novel electrochemical sensing platform consisting of an aminated zeolite imidazolium ester backbone material (ZIF-8-NH2) composited with sodium oxidized alginate (OSA)/carboxymethyl chitosan (CMCS) dynamic hydrogel (denoted as OC hydrogel) was successfully constructed. The platform exploits the mild Schiff base reaction between OSA and CMCS to construct a three-dimensional dynamic hydrogel substrate without an external cross-linking agent. On this basis, the combination of ZIF-8-NH2 and OC hydrogel was introduced. The amino groups on the surface of ZF-8-NH2 and the aldehyde groups of OSA are utilized to form covalent nano-crosslinking points, simultaneously exerting their electrocatalytic activity and pre-enrichment effect.

**Results:**

Electrochemical studies demonstrated that the composite interface significantly accelerated electron transfer kinetics. The constructed sensor demonstrates exceptional analytical performance for hydroquinone. The detection limit is as low as the nanomolar level (16.7 nM), and the linear range extends up to four orders of magnitude (0.05 to 1800 μM). Furthermore, the sensor demonstrated its efficacy in the rapid analysis of a wide range of real samples (tap water, drinking water, beverages, and vegetable juices), with recoveries ranging from 97.4 to 103.6%.

**Discussion:**

The observed performance is attributed to the multiple synergistic effects of the ZIF-8-NH2/OC hydrogel interface. The covalent connection between ZIF-8-NH2 and the OSA/CMCS hydrogel prevents nanomaterial detachment, ensuring interface stability. Meanwhile, ZIF-8-NH₂ reduces the overpotential for hydroquinone electrochemical oxidation and achieves significant signal amplification through pre-enrichment of target molecules. These features substantiate the reliability of the sensor in practical applications, offering an innovative solution for the detection of harmful substances in food.

## Graphical abstract

**Figure:**
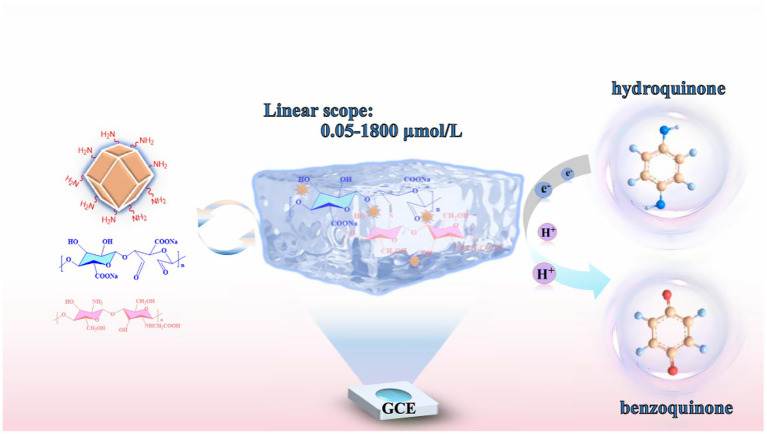
This study has developed a synergistic enrichment and catalytic sensing platform based on functionalized ZIF-8 and dynamic Schiff base hydrogels, enabling ultrasensitive detection of hydroquinone in foodstuffs.

## Highlights

- Hydrogel was synthesized through a green, mild Schiff reaction that does not necessitate external crosslinking agents.
- ZIF-8-NH₂ exhibits high catalytic activity and dispersibility in the gel substrate.
- This sensor has outstanding performance in terms of high recovery rate, good stability and anti-interference ability for real samples.

## 1 Introduction

The issue of food safety represents a significant national concern. Hydroquinone (HQ) is a phenolic compound that has attracted significant attention due to its role as a residue problem in food (1). HQ has the potential to be neurotoxic and genotoxic, and its safety assessment in organizations such as the Joint Expert Committee on Food Additives (JECFA) is extremely stringent (2, 3). Therefore, it is crucial to establish a rapid and highly sensitive method for the detection of HQ in food. At present, the detection of phenolics is chiefly dependent on high performance liquid chromatography (HPLC) and gas chromatography–mass spectrometry (GC–MS) (4, 5). Despite the sensitivity and accuracy of these methods, they are reliant on large-scale instruments, complicated pre-treatment, high detection costs and long time-consuming processes, which are difficult to meet the urgent needs of real-time monitoring in the field. Electrochemical sensing technology boasts a number of significant advantages, including rapidity, portability and low cost, and shows great potential for application in the field of food safety monitoring (6).

The core of high-performance electrochemical sensors lies in the modifying materials at the electrode interface. In recent times, considerable attention has been focused on hydrogels within the domain of electrochemical biosensors, owing to their superior biocompatibility, remarkable adhesion properties, and capacity to establish conformational contacts with diverse surfaces (7). Oxidized sodium alginate (OSA)/carboxymethyl chitosan (CMCS) hydrogel, as an emerging functional material (8, 9), shows significant advantages in constructing high-performance electrochemical sensors. The Schiff base reaction between the aldehyde group introduced to the molecular chain of oxidized sodium alginate and the amino group on the chain of carboxymethyl chitosan can spontaneously form a three-dimensional network structure at room temperature and neutral pH, without the need to introduce exogenous toxic cross-linking agents (e.g., glutaraldehyde) or apply external stimuli such as ultraviolet light and high temperature (10). This method effectively avoids the possible problem of the traditional chemical cross-linking agents that may cause biomolecular inactivation problems. Furthermore, the hydrophilic groups (e.g., carboxyl, hydroxyl) that are abundant in the hydrogel network ensure sufficient hydration (11). This in turn provides efficient channels for the diffusion of electroactive substances and the migration of ions, thus facilitating a rapid current response. It is important to note that the aldehyde group of OSA and the amino and carboxyl groups of CMCS facilitate self-cross-linking, while also functioning as covalent anchors for immobilizing more complex recognition elements or directly coupling conductive nanomaterials (12). Nevertheless, when confronted with the task of detecting complex water samples, a solitary hydrogel material confronts several obstacles. These include a restricted specific surface area, a mass transfer rate constrained by the gel network, and inadequate target enrichment, which curtails the potential for enhancement of its detection sensitivity.

In order to overcome the aforementioned limitations, the integration of functional nanomaterials into hydrogel matrices to engineer composite sensing interfaces has emerged as a viable approach (13). Metal–Organic Frameworks (MOFs) are a class of crystalline porous materials formed by the self-assembly of metal ions with organic ligands. These materials have attracted widespread attention for their extremely high specific surface area, tunable pore size and rich chemical diversity (14–16). Their incorporation into hydrogel composites represents a judicious approach to further enhance the catalytic activity of electrode materials. However, research into MOF-based hydrogels for electrochemical detection remains scarce at present, and there are several key problems that need to be solved urgently (17–19). Taking the existing research as an example, Chong et al. physically embedded Zr-MOF into chitosan/alginic acid hydrogel for the detection of chlorogenic acid. Although they achieved a wide linear range and a low detection limit, the weak interfacial binding force resulted from the physical mixture between the MOF and the hydrogel led to insufficient long-term stability (18). Similarly, Sun et al. combined MOF/COF core-shell composite materials with chitosan-based hydrogels to construct an efficient sensing platform for the portable detection of desmopressin tartrate. However, their system still relied mainly on physical composites, which not only had a complex preparation process but also failed to achieve covalent bonding between the MOF and the hydrogel (19). Thus, it can be seen that how to achieve stable interfacial binding between nanomaterials and hydrogels, while simplifying the preparation process, remains a bottleneck that needs to be overcome in this field.

Zeolite imidazolium ester skeleton material-8 (ZIF-8) is a typical member of the family of MOFs. It has the advantages of ease of synthesis and good chemical stability (20). However, the original ZIF-8 is hydrophobic and lacks active sites for robust binding to polymeric matrices. The introduction of aminated derivative ZIF-8-NH₂ can effectively address the aforementioned issues. The incorporation of amino groups not only enhances interfacial stability through covalent cross-linking with the gel network via Schiff base reactions, but also improves the material’s hydrophilicity and dispersibility (21). This prevents particle agglomeration and fully exposes the active surface. Furthermore, the electron-rich amino group contributes to elevated catalytic activity and selective adsorption capacity. This design effectively makes up for the shortcomings of the existing MOF/ hydrogel materials, such as weak interface bonding and cumbersome preparation process, providing a new idea for the construction of high-performance MOF/ hydrogel sensing platforms.

**SCHEME 1:**
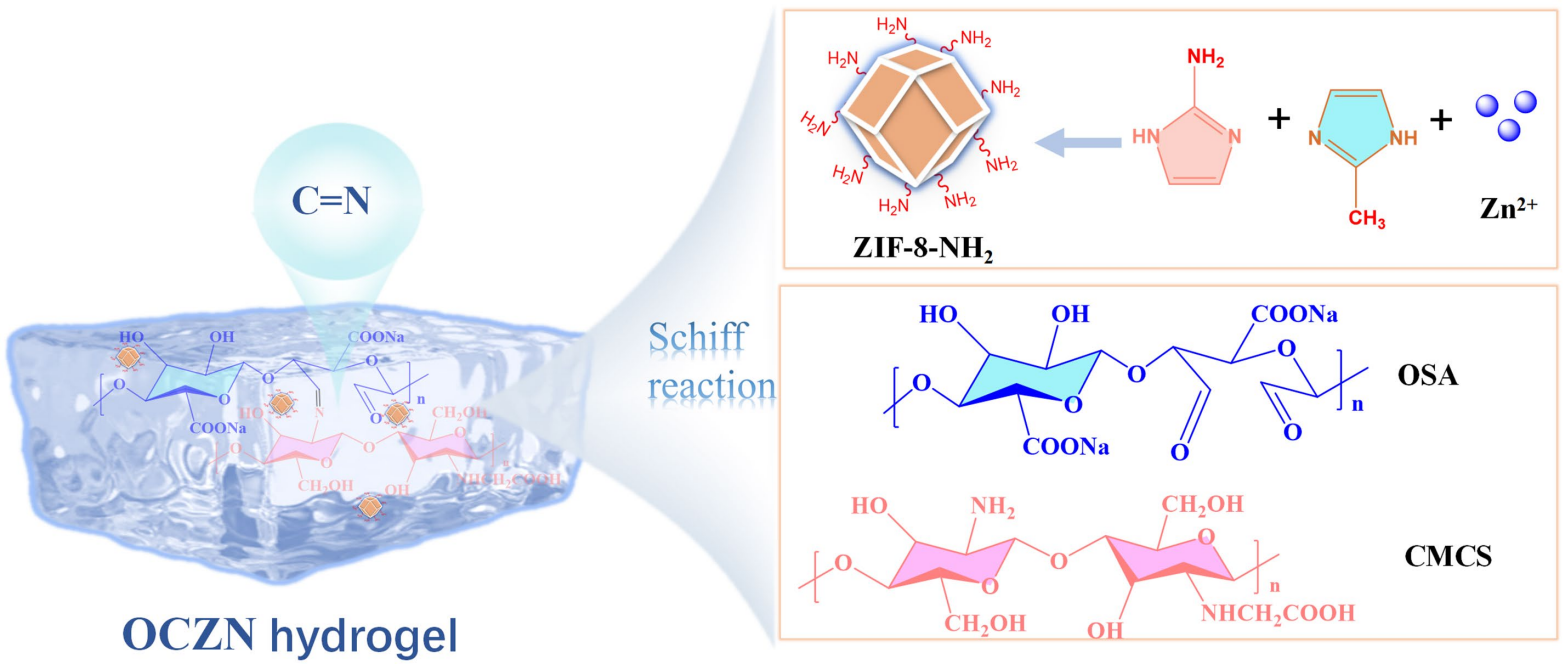
Synthesis diagram of OCZN hydrogel.

In this study, a high-performance, dynamically cross-linked hydrogel was designed and constructed. In particular, CMCS and OSA form a hydrogel network through dynamic imine bonding, while amine-functionalized MOF is anchored to the hydrogel network through covalent cross-linking with OSA (Scheme 1). Furthermore, the -NH_2_ group present on the surface of ZIF-8-NH_2_ has been shown to promote electron transfer between the interface of HQ and the electrode through the hydrogen bonding network, thereby significantly enhancing the enrichment efficiency of target molecules. The utilization of the sensor for the quantitative analysis of HQ in tap water and beverages yielded satisfactory recoveries (96.3–104.7%), thereby demonstrating its potential for practical implementation in food environmental monitoring. To the best of our knowledge, this is the first report on the use of amino-functionalized ZIF-8 as a covalent nano-crosslinker, which is chemically integrated with OSA/CMCS hydrogel through dynamic Schiff base bonds to construct a multifunctional sensing interface. Furthermore, we demonstrate its application in the highly sensitive detection of hydroquinone.

## 2 Materials and methods

### 2.1 Chemicals

Analytical reagents, abbreviated as AR, Nitric acid hexahydrate (Zn(NO_3_)_2_ ·6H_2_O, AR), were supplied by China Shanghai Sinopharm Chemical Reagent Co. The following reagents were procured from the Aladdin Chemical Reagent Company, Shanghai, China: carboxymethyl chitosan (CMCS), sodium alginate (SA), sodium periodate, disodium hydrogen phosphate dodecahydrate (Na_2_HPO_4_ ·12H_2_O, AR), sodium phosphate dihydrate (NaH_2_PO_4_ ·2H_2_O, AR) and 2-methylimidazole (C_4_H_6_N_2_, 98%). Phosphate buffer solution (PBS) was selected as the support electrolyte, and PBS was meticulously prepared with 0.1 M of NaH_2_PO_4_ and 0.1 M of Na_2_HPO_4_ in various ratios. To obtain deionised water, the Millipore water purification system was employed. With the exception of deionised water, all chemicals were utilized in their original state, without undergoing any additional purification processes.

### 2.2 Apparatus

The morphology of the samples was analyzed on a Hitachi S-4800II field emission scanning electron microscope (FESEM). The Fourier transform infrared spectroscopy (FT-IR) was conducted using a Cary 610/670 infrared microspectrometer from Varian, USA. X-ray diffraction (XRD) maps in the 2θ range 5–80° were recorded with a D8 Advance X-ray diffractometer from Bruker (Germany). X-ray photoelectron spectroscopy (XPS) was obtained with an ESCALAB 250Xi XPS spectrometer (equipped with an aluminium Kα X-ray source) from Thermo Scientific (USA). Water contact angles (CAs) were measured by means of an optical contact angle meter (JC2000D, Shanghai Zhongchen Digital Technology Instrument Co., Ltd.). The present study employed high performance liquid chromatography (HPLC, LC-20A) in conjunction with a Shimadzu (Kyoto, Japan) chromatograph and UV/vis detector (SPD-20 AV). The electrochemical evaluation of all modified electrodes was carried out on an electrochemical analyzer (CHI852C, CH Instruments Co., Shanghai, China) using a high sensitivity detection system (three-electrode device). The electrochemical performance analysis incorporated the prepared material-modified glassy carbon electrode (GCE) as the working electrode, platinum (Pt) wire electrode as the counter electrode, and silver/silver chloride (Ag/AgCl) electrode as the reference electrode.

### 2.3 Preparation of ZIF-8 and ZIF-8-NH_2_

The synthesis of ZIF-8 and ZIF-8-NH_2_ was carried out according to the literature (22): 2.975 g of zinc nitrate hexahydrate and 3.284 g of 2-methylimidazole were dissolved in 100 mL of methanol, respectively. Then zinc nitrate solution was added to 2-methylimidazole solution and stirred vigorously for 1 h. Finally, the sample (ZIF-8) was washed with methanol for 3 times, and dried under vacuum at 60 °C for 8 h. As for ZIF-8-NH_2_, 1.488 g of Zn(NO_3_)_2_·6H_2_O was dissolved in 50 mL of DMF. 0.270 g of 2-aminobenzimidazole, 1.478 g of 2-methylimidazole, and 0.1 g of sodium formate were added in 50 mL of deionised water, and heat it up at 70 °C for 2 h. Then the zinc nitrate solution was added into the above mixture and stirred for 30 min. The obtained material (ZIF-8-NH_2_) was centrifuged for 10 min (1,100 rpm), and washed with methanol for three times, and dried in vacuum at 60 °C for 8 h.

### 2.4 Preparation of oxidized sodium alginate (OSA)

Five gram of SA was dissolved in 200 mL of deionised water solution. After dissolution, 4 g of sodium periodate was added under stirring for 5 h at room temperature, 5 mL of ethylene glycol was added to terminate the reaction. After 30 min, the solution was dialyzed in deionized water for 3 days, and OSA was obtained after freeze-drying for 72 h (23).

### 2.5 Preparation of OC hydrogel and OCZN composite hydrogel

A 2% sodium oxyalginate (OSA) solution and a 5% carboxymethyl chitosan (CMCS) solution were mixed at room temperature in a volume ratio of 1:2, and then cross-linked to form OSA/CMCS hydrogel (OC). Then, 20, 30 and 40 mg of ZIF-8-NH_2_ were, respectively, added to 10 mL of 5% CMCS solution, and ultrasonically dispersed uniformly to prepare a series of CMCS/ZIF-8-NH_2_ mixed dispersions. Subsequently, each dispersion was mixed with 2% OSA solution at a volume ratio of 1:2 at room temperature and cross-linked. Eventually, three composite hydrogels with different ZIF-8-NH_2_ loading capacities were obtained, which were named OCZN-20, OCZN-30 and OCZN-40 in sequence.

### 2.6 Electrode preparation

The electrode underwent ultrasonic cleaning in deionized water and ethanol, then air-dried under nitrogen. A 5 μL droplet of OCZN-30 hydrogel suspension was applied to the GCE surface and allowed to air-dry at room temperature, yielding the OCZN-30/GCE modified electrode. The same procedure was employed to prepare ZIF-8-NH_2_/GCE, OC/GCE, OCZN-20/GCE, OCZN-30/GCE, and OCZN-40/GCE electrodes. The modified electrodes were stored in a desiccator at room temperature.

## 3 Results and discussion

### 3.1 Characterization of prepared materials

**Figure 1:**
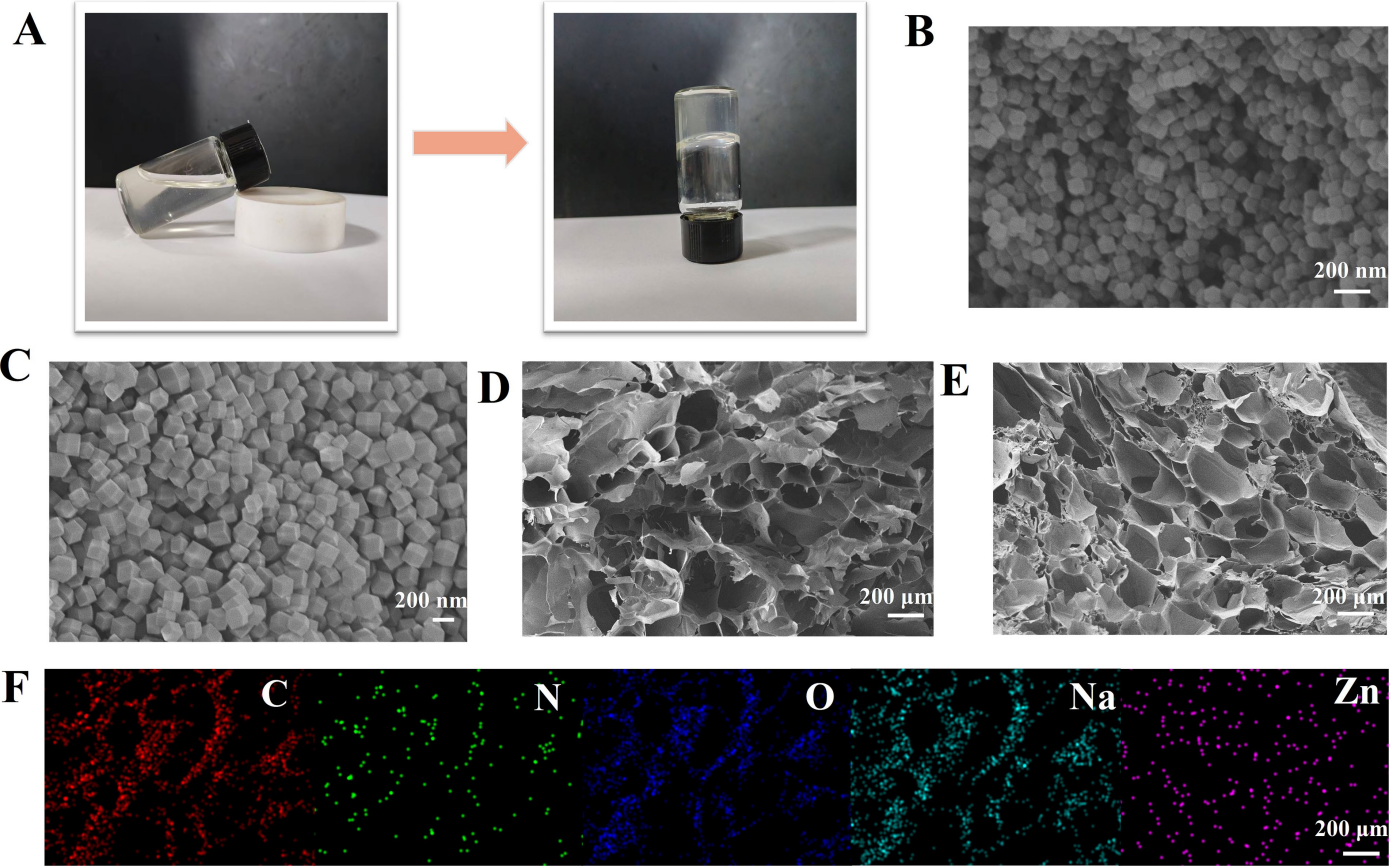
**(A)** Schematic diagram of sol–gel transition behavior. SEM images of ZIF-8 **(B)**, **(C)** ZIF`-8-NH_2_, **(D)** OC, and **(E)** OCZN, **(F)** Spectral element mapping of OCZN.

As shown in Figure 1A, when CMCS/ZIF-8-NH₂ solution is mixed with OSA solution, the liquid changes from a flowing state to a gel-like state. When the container is inverted, it does not flow and shows a stable gel-like state. Then, the morphology of the internal constituent substances of the hydrogel was observed by SEM. As demonstrated in the accompanying Figure 1B, ZIF-8 displays a rhombic dodecahedral structure. ZIF-8-NH₂ (Figure 1C) exhibits a morphology consistent with that of ZIF-8 (24), yet possesses augmented dimensions, suggesting that amination did not compromise the integrity of the ZIF-8 framework. As illustrated in Figure 1D, the SEM images of the OC hydrogels demonstrate the presence of a cross-linked porous structure, which is primarily attributed to the formation of Schiff base bonds and chain entanglement between OSA and CMCS. Upon the incorporation of MOF (Figure 1E), a decline in the pore size of the OCZN hydrogel network was observed, resulting in a more compact and dense network structure. This phenomenon can be attributed primarily to the formation of Schiff base bonds between the amino group on ZIF-8-NH_2_ and the hydrogel, accompanied by an augmentation in crosslink density. Energy dispersive X-ray spectroscopy (Figure 1F) analysis of the hydrogels demonstrated that elemental zinc (Zn) was distributed uniformly within the porous structure of the OCZN hydrogels, thereby confirming the uniform doping of ZIF-8-NH_2_ into the hydrogels.

**Figure 2:**
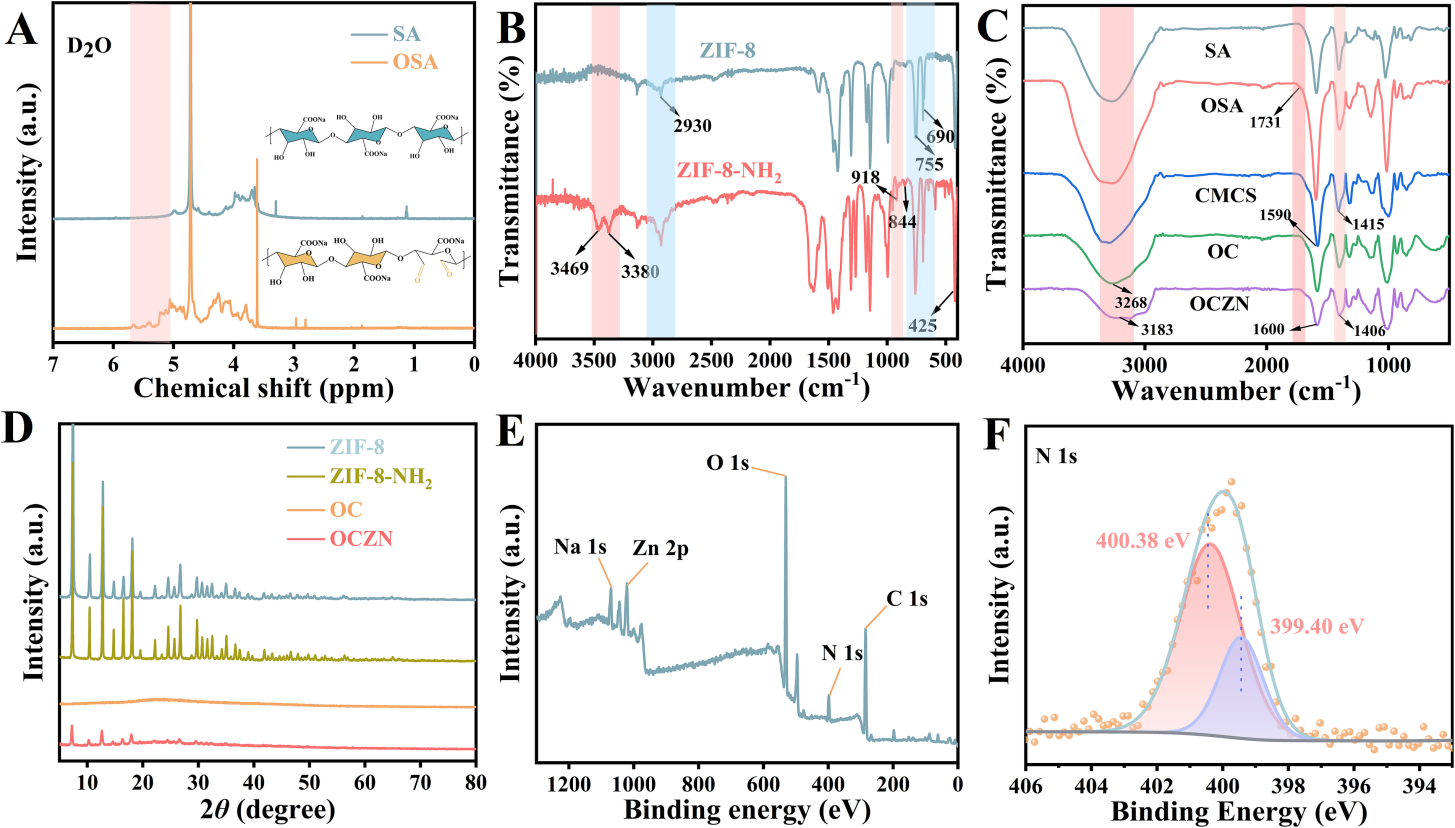
**(A)** ^1^H NMR spectra of SA and OSA. **(B)** FTIR spectra of ZIF-8 and ZIF-8-NH_2_. **(C)** FTIR of intermediate products and freeze-dried hydrogels. **(D)** XRD of ZIF-8, ZIF-8-NH_2_, OC, and OCZN hydrogels. **(E)** XPS survey spectrum of OCZN. **(F)** N 1 s XPS spectra of OCZN.

As illustrated in Figure 2A, In the nuclear magnetic resonance hydrogen spectroscopy (^1^H NMR) analysis, the OSA spectra showed a characteristic signal peak not observed in the SA spectra within the *δ* 5.25–5.75 ppm chemical shift range, which belonged to the hemiacetal structure formed during the oxidation process (25). The results confirmed the successful oxidation modification of sodium alginate. The successful preparation of materials was determined by FTIR spectroscopy. As demonstrated in Figure 2B, the characteristic peaks attributed to ZIF-8 occurred at 2930, 755, 690, and 425 cm^−1^, indicating the successful preparation of ZIF-8 (26). The characteristic peaks in the infrared spectrum of ZIF-8-NH_2_ are similar to those of ZIF-8. The only difference is that the characteristic peaks at 3470 and 3,385 cm^−1^ belong to the -NH_2_ group, while the peaks at 918 and 844 cm^−1^ belong to the -NH group in 2-aminobenzimidazole (27), confirming the successful introduction of amino groups in ZIF-8 and indicating the successful preparation of ZIF-8-NH_2_ nanoparticles. As demonstrated in Figure 2C, both SA and OSA manifest a wide absorption peak within the range of 3,685–2,875 cm^−1^, which is ascribed to the stretching vibration of the intramolecular hydroxyl group (O-H) (28). It is noteworthy that, in comparison with SA, OSA exhibited a novel characteristic peak at 1731 cm^−1^, attributable to the vibrational absorption of the C=O bond within the aldehyde group (29). This peak is relatively weak, possibly due to the formation of hemiacetal, which hinders the detection of free aldehydes (30). This finding aligns with the results of ^1^H NMR. The broad peak of CMCS in the range of 3,680–2,970 cm^−1^ corresponds to the superposition of stretching vibrations of the amino group (N-H) and the hydroxyl group (O-H). The characteristic peaks located at 1590 cm^−1^ and 1,415 cm^−1^, respectively, belong to the stretching vibration of the amino group (-NH₂) or the asymmetric stretching vibration of the carboxyl group (COO^−^) and the symmetrical stretching vibration of the carboxyl group.

In the infrared spectrum of the OC hydrogel, the disappearance of the carbonyl characteristic peak at 1731 cm^−1^ in OSA is clearly evident, along with a significant weakening of the amino absorption peak near 1,590 cm^−1^. These changes indicate that a Schiff base reaction has occurred between the aldehyde group on the OSA molecular chain and the amino group on the CMCS molecular chain (31). Furthermore, following the introduction of ZIF-8-NH₂ into the system, the absorption peak intensity at 1600 cm^−1^ exhibited a further weakening, and the N–H/O–H stretching vibration peak near 3,268 cm^−1^ demonstrated a red shift. This indicates that the amino groups on the surface of ZIF-8-NH₂ might also have participated in the Schiff base reaction, and there might be hydrogen bond interactions between it and the polymer network.

Moreover, the XRD analysis results in the Figure 2D show that the characteristic diffraction peaks of ZIF-8-NH₂ remain consistent with those of ZIF-8 (32), indicating that the crystal structure of ZIF-8-NH₂ has not undergone significant changes due to the amino modification, which confirms the successful synthesis of ZIF-8-NH₂. The XRD pattern of the pure OC hydrogel exhibits typical amorphous diffuse peaks, while in the pattern of the OCZN composite hydrogel, the characteristic diffraction peaks of ZIF-8-NH₂ can be observed, although their intensity is weakened due to the encapsulation effect of the polymer matrix. This result further confirms that ZIF-8-NH₂ has been successfully loaded into the hydrogel system. According to the XPS survey spectrum, the OCZN hydrogel is composed of C, N, O, Na, and Zn elements (Figure 2E). The high-resolution XPS spectra are shown in Figure 2F. The two peaks of N 1 s at 399.40 and 400.38 eV are assigned to C=N bond, and -N-H, respectively (33). This signal directly confirmed that a Schiff base reaction occurred between the carboxymethyl chitosan molecule and the amino group in ZIF-8-NH_2_ and the aldehyde group on the oxidized sodium alginate chain, forming a dynamic imine bond, thereby constituting the three-dimensional network skeleton of the hydrogel.

### 3.2 The physical properties of hydrogels materials

**Figure 3:**
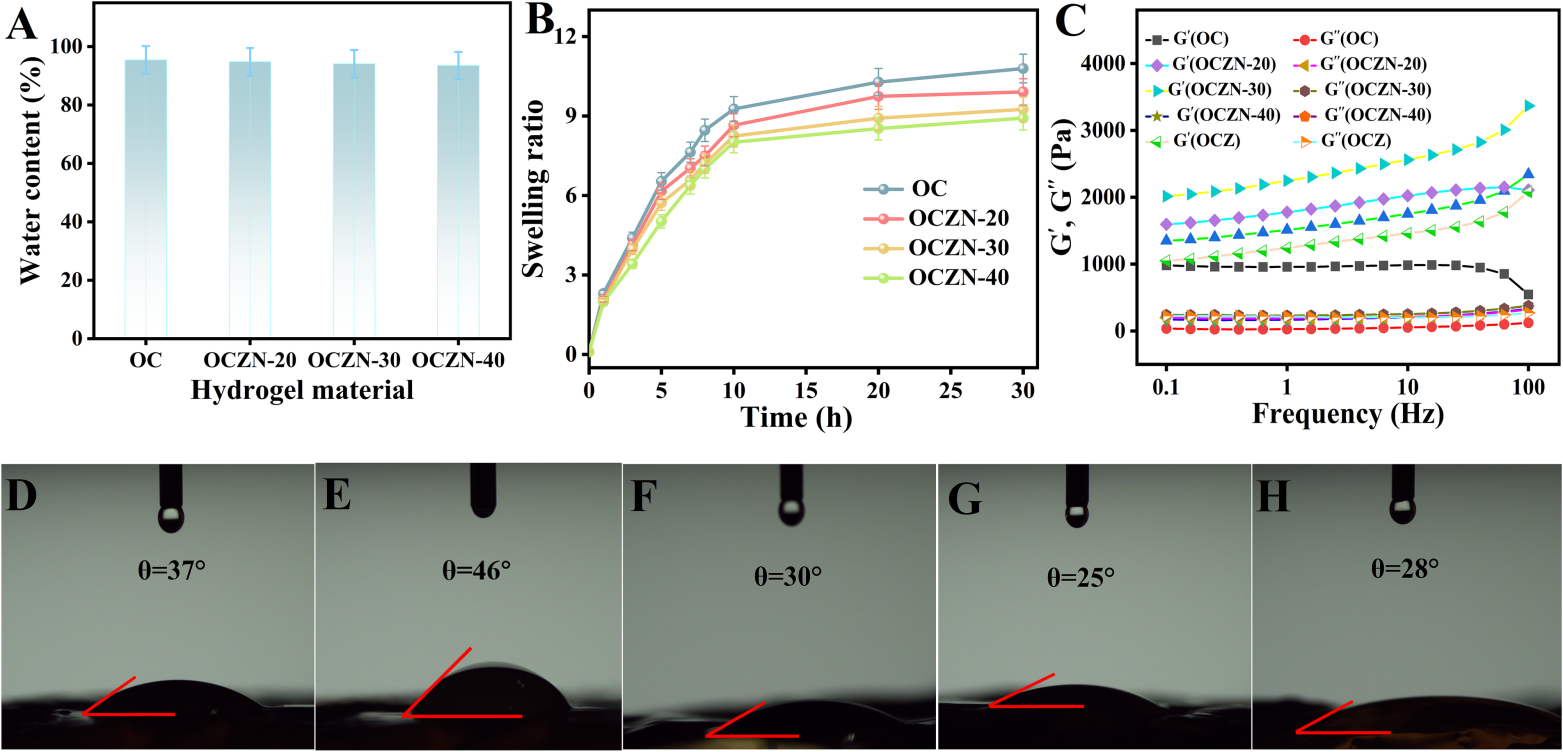
Water content **(A)** and swelling ratio **(B)** of OC, OCZN-20, OCZN-30, and OCZN-40 hydrogels. **(C)** The energy storage modulus (G’) and loss modulus (G”) vary with the frequency of OC, OCZN-20, OCZN-30, OCZN-40, and OCZ hydrogels. Water contact angle of **(D)** OC, **(E)** OCZ, **(F)** OCZN-20, **(G)** OCZN-30, and **(H)** OCZN-40 hydrogels.

The physical properties of hydrogels are related to the mass transfer efficiency of analytes and thus affect the electrochemical performance of sensors (34). Therefore, it is necessary to conduct research on them. As illustrated in the Figure 3A, the water content of each group of hydrogels can exceed 90%. Appropriate swelling properties are crucial for electrochemical sensing to balance ion transport, structural stability and interfacial reliability. As shown in the Figure 3B, the dried hydrogels were dissolved in deionized water, and the results obtained demonstrated that all of the hydrogels exhibited high water absorption and rapid swelling over the initial 6 h period. The swelling equilibrium was achieved within 10 h. It is noteworthy that the swelling rate of OC hydrogel was higher than that of OCZN hydrogel loaded with ZIF-8-NH_2_. Furthermore, it was observed that the higher the loading, the lower the swelling rate. This phenomenon can be attributed to the specific characteristics of ZIF-8-NH_2_, which occupies a portion of the pores within the three-dimensional network of the hydrogel. The crosslinked network of OCZN is denser, which impedes the entry of water molecules into the hydrogel interior, consequently reducing its swelling. Furthermore, the storage modulus and loss modulus of the individual hydrogels were analyzed using a rotational rheometer (Figure 3C). The results demonstrated that the storage modulus (G’) was greater than the loss modulus (G”) in all materials, which exhibited solid-state gel properties (35). It is worth noting that compared with OCZ hydrogels, the G’ of OCZN is higher. This can be attributed to the fact that the ZIF-8-NH₂ of OCZN significantly increases the crosslinking density of the hydrogel network through covalent crosslinking, thereby endowing it with superior mechanical strength and structural stability.

The electrochemical response is contingent on the adsorption of electrolyte ions on the surface; thus, enhancing surface wettability would be advantageous for improving the sensing performance of electrochemical sensors (36). Dynamic water contact angle measurements were performed on individual hydrogels. As demonstrated in Figures 3D–H, the OCZN hydrogels exhibits higher hydrophilicity, and its water droplet adsorption is significantly better than that of OC hydrogel. In contrast, due to the hydrophobicity of ZIF-8, the contact angle of OCZ hydrogel is the largest. This finding indicates that the incorporation of ZIF-8-NH_2_ can enhance the surface hydrophilicity of the OC hydrogels. This is attributable to the presence of a substantial number of polar groups, such as -NH_2_ and -OH, on the surface of ZIF-8-NH_2_. These groups possess the capacity to form hydrogen bonds with water molecules and thereby reduce the surface free energy, thus augmenting the wettability.

### 3.3 Electrochemical behavior of different modified electrodes

**Figure 4:**
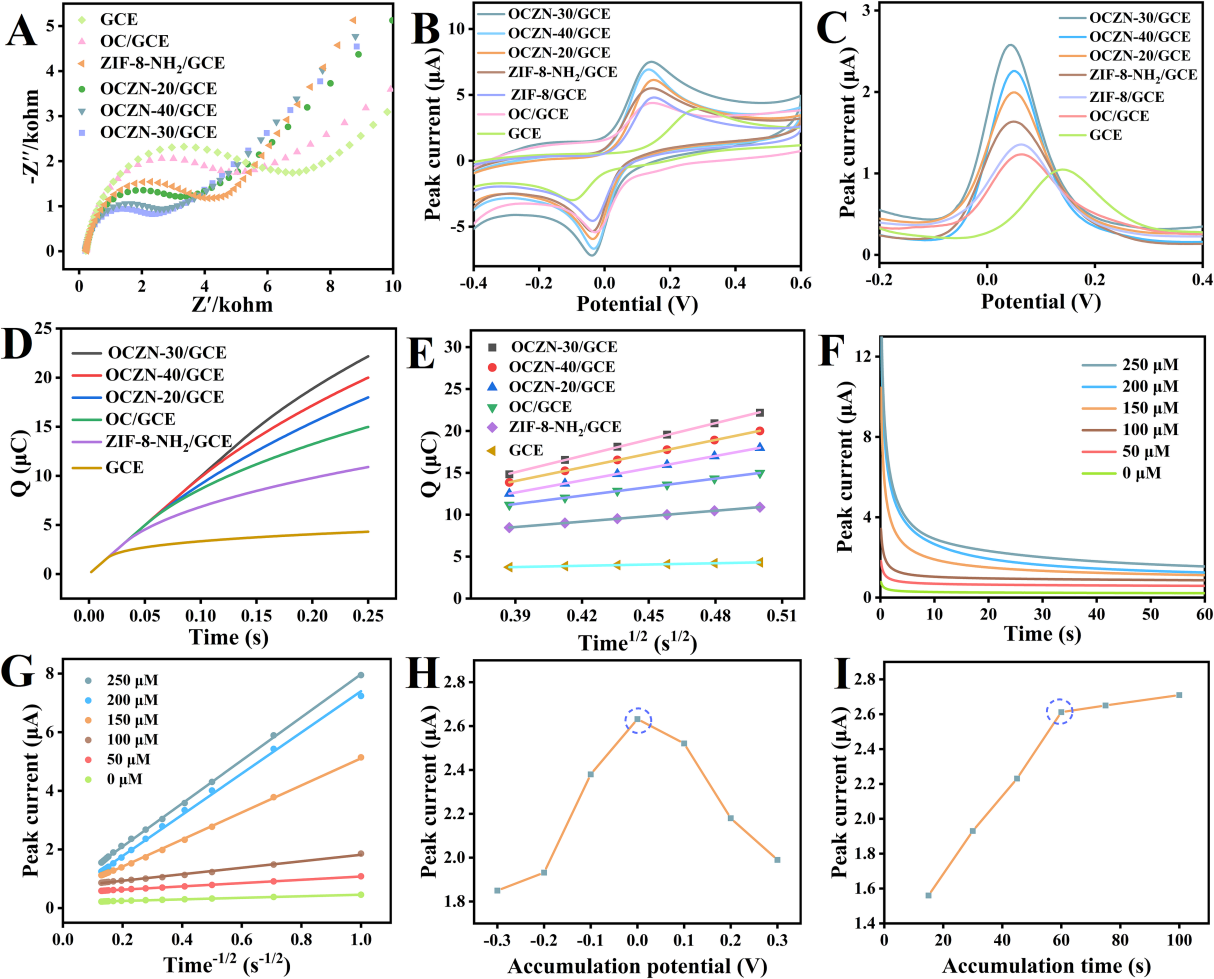
**(A)** EIS in 5.0 mM [Fe(CN)_6_]^3−/4-^ containing 0.1 M KCl for GCE, OC/GCE, ZIF-8-NH_2_/GCE, OCZN-20/GCE, OCZN-30/GCE, and OCZN-40/GCE. **(B)** CV and **(C)** DPV curves of 100 μM HQ in PBS for GCE, OC/GCE, ZIF-8/GCE, ZIF-8-NH_2_/GCE, OCZN-20/GCE, OCZN-30/GCE, and OCZN-40/GCE. **(D)** Chronocoulometry curves of three electrodes in 1.0 mM [Fe(CN)_6_]^3−/4-^ containing 0.1 M KCl. **(E)** The linear relationship between *Q* and *t*^1/2^ derived from chronocoulometry data. **(F)** Chronoamperometric response at the OCZN-30 in 0.1 M PBS with varied HQ concentrations: 0, 50, 100, 150, 200, and 250 μM. **(G)** The linear relationship between*I*and *t*^-1/2^ derived from the chronoamperometric data. **(H)** Line diagram of 100 μM HQ detection in 0.1 mol/L PBS solution by accumulated potential; **(I)** Line chart of 100 μM HQ detection in 0.1 mol/L PBS solution during accumulation time.

The electron transfer kinetics of the different modified materials were evaluated by electrochemical impedance spectroscopy measurements. The Nyquist plots consisted of semicircles at high frequencies and straight lines at low frequencies, thus representing typical features of charge transfer (R_ct_) and diffusion confinement processes, respectively (37). Electrical impedance spectroscopy (EIS) analyses were carried out in 5 mM [Fe(CN)_6_]^3−/4-^solution (containing 0.1 M KCl) to explore electron transfer processes at different electrodes. As demonstrated in Figure 4A, OCZN-30/GCE exhibited the lowest charge transfer resistance, followed by OCZN-40/GCE, OCZN-20/GCE, OCZN-ZIF-8-NH_2_/GCE, OC/GCE, and bare GCE, thus confirming its superior charge transfer kinetics, which facilitates electron transfer from the electrode surface to the electrolyte.

The use of cyclic voltammetry (CV) and differential pulse voltammetry (DPV) was employed to facilitate an examination of the electrochemical disparities amongst a range of modified electrodes for 100 μM HQ, as illustrated in Figures 4B,C. The bare electrode exhibited a less pronounced peak at elevated potentials. However, the peak potential diminished and the peak current augmented subsequent to the modification of the material. This observation signifies that the modified material possesses augmented catalytic activity, thereby reducing the activation energy barrier of the reaction and expediting the electron transfer. This is attributed to the unique structure of ZIF-8-NH₂, where the Zn-N sites may act as Lewis acid centers to activate the HQ molecule, while the adjacent surface amino groups (-NH₂) may stabilize the oxidation reaction intermediates through hydrogen bond interactions, thereby jointly reducing the electron transfer barrier (38). Therefore, the ZIF-8-NH₂ modified interface significantly reduces the overpotential of HQ oxidation and exhibits excellent electrocatalytic activity. In the case of the various modified electrodes, OCZN-30/GCE demonstrated a significantly more pronounced peak current response in comparison to the others. The results of the above DPV experiments show a significant negative correlation with the EIS data. Specifically, the R_ct_ of the OCZN-30 modified electrode decreased significantly from 6.2 kΩ of the bare GCE to 2.6 kΩ, while the DPV peak current increased from 1.1 μA to 2.64 μA, approximately enhancing by 1.4 times. This quantitative consistency confirms that the reduction in R_ct_ (i.e., the acceleration of interface electron transfer kinetics) is the direct cause of the enhanced DPV signal. These results collectively demonstrate that OCZN-30 is an excellent modification material that can significantly improve the interface electron transfer kinetics and electrocatalytic performance.

In a 10 mL solution of 5 mM [Fe(CN)_6_]^3−/4-^ (containing 1.0 M KCl), the chronocoulometry was utilized to ascertain the electrochemically active surface area (ECSA) of diverse sensing interfaces (Figure 4D). The total charge (Q) of each modified electrode exhibited a linear relationship with the square root of time (t^1/2^), as shown in the Figure 4E. by the Anson formula (39): *Q* = 2*nFACD*^1/2^*π*^-1/2^*t*^1/2^ + *Q*_dI_ + *Q*_ads_, where *n* denotes the [Fe(CN)_6_]^3−/4-^ electron transfer number (*n* = 1), *F* represents the Faraday constant (96485.33 C/mol), *C* signifies the concentration of [Fe(CN)_6_]^3−/4-^ (1 mM), and *D* denotes the diffusion coefficient of [Fe(CN)_6_]^3−/4-^(6.7 × 10^−6^ cm^2^·s^−1^). *Q*_dI_ and *Q*_ads_ denote the bilayer charge and the Faraday charge generated by the oxidation of the analyte, respectively. The ECSAs were calculated to be 0.172, 0.319, 0.395, 0.412, 0.489, and 0.451 cm^2^ for GCE, OC/GCE, ZIF-8-NH_2_/GCE, OCZN-20/GCE, OCZN-30/GCE, and OCZN-40/GCE, respectively. Among them, the ECSA of OCZN-30/GCE was the highest, indicating that it can provide more active sites for HQ catalysis and accelerate its chemical reaction speed on the electrode surface, thus improving the sensitivity of detection.

The diffusion coefficient is a pivotal parameter that describes the diffusion behavior of an analyte in solution. The present study investigates the diffusion coefficient (D) of HQ in the electrochemical system. The investigation is conducted using the chronoamperometry method, with OCZN-30/GCE serving as the electrochemical sensor (Figure 4F). Based on the data in Figure 4G and the Cottrell equation (40): *I* = *nFAD*^1/2^π^-1/2^ *t*^-1/2^*C*, here, *n* and *C* stand for the number of electron transfers and the initial concentration of HQ, respectively. The term *A* denotes the electrochemically active surface area of OCZN-30/GCE. The present study sought to calculate the diffusion coefficients of HQ at varying concentrations (50, 100, 150, 200 and 250 μM). The results indicate that the diffusion coefficients are 7.824 × 10^−7^, 6.291 × 10^−7^, 5.732 × 10^−7^, 5.013 × 10^−7^ and 3.804 × 10^−7^ cm^2^ s^−1^, respectively. It should be noted that these are apparent diffusion coefficients influenced by interfacial adsorption, rather than the intrinsic diffusion coefficients of HQ (41). The strong adsorption capacity of ZIF-8-NH₂ at the OCZN-30/GCE interface forms a concentrated adsorption layer, which disrupts the Cottrell equation assumption. The findings suggest that higher concentrations increase the resistance to mass transfer, which in turn affects the analyte to electrode surface diffusion rate.

### 3.4 Optimization of experimental conditions

In order to enhance the sensitivity of HQ detection using the OCZN-30/GCE electrode, it was necessary to optimize key parameters such as accumulation potential, accumulation time and PBS pH. As demonstrated in the accompanying Figure 4H, the potential range of −0.3 to 0.3 V was investigated for its effect on the peak HQ current. The peak current of 100 μM HQ reached its maximum value at 0 V, indicating the optimal HQ accumulation on the OCZN-30/GCE surface. Consequently, 0 V was identified as the optimal accumulation potential for effective HQ detection. For the 100 μM HQ solution, the accumulation time was varied between 15 s and 90 s. The effect of deposition time was evaluated as shown in Figure 4I. The peak current gradually increased with the accumulation time, and an accumulation time of 60 s was chosen for further measurements in order to balance the sensitivity with the analysis time, which ensured efficient analyte collection.

**Figure 5:**
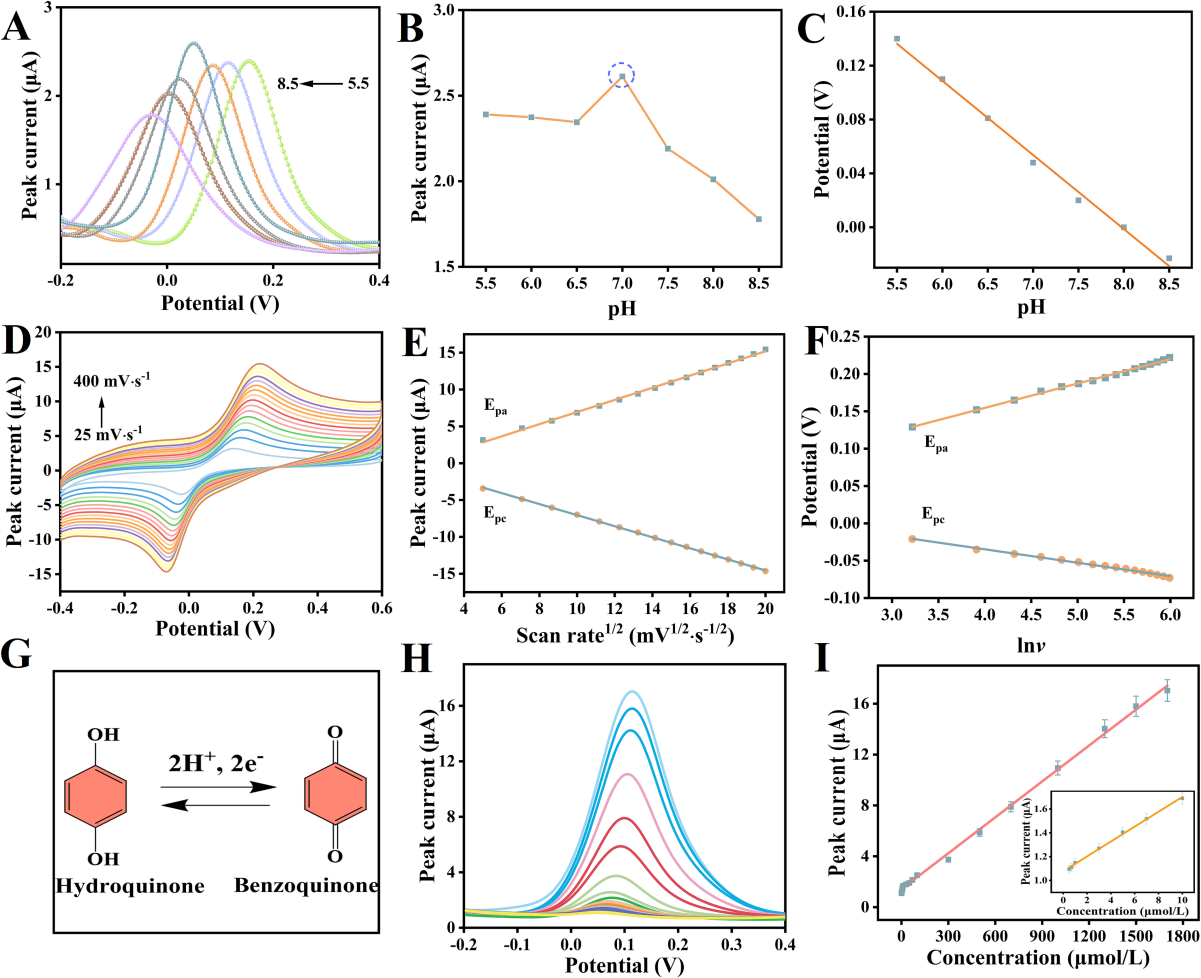
**(A)** The influence of electrode material modification amount on the oxidation peak current of 100 μM HQ. **(B)** The influence of different pH (5.5–8.5) on the peak current of HQ (the illustration shows a line chart of the relationship between different pH and the corresponding peak current). **(C)** The linear relationship between different pH and potential. **(D)** CV curves for 100 μM HQ at the OCZN-30/GCE in 0.1 M PBS (pH = 7.0) at different scan rates (from 25 and 400 mV·s^−1^). **(E)** The dependence of redox peak currents on the square root of different scanning rates. **(F)** The linear relation between the *E*pa and *E*pc of HQ and ln *ν*. **(G)** The electrochemical redox mechanism of HQ detected by OCZN-30/GCE. **(H)** Current response diagram of OCZN-30/GCE to HQ at different concentrations (0.05–1800 μM); **(I)** Linear plot of current versus HQ concentration (the illustration shows a linear graph ranging from 0.05 to 10 μM).

To investigate the influence of the thickness of the OCZN-30 hydrogel layer on the sensing performance, we systematically optimized the drop coating volume (1,3, 5, 7, 9 μL). As shown in the Figure 5A, with the modification amount increasing from 1 μL to 5 μL, the peak current of DPV significantly increased, attributed to more active sites being introduced into the interface, which enhanced the pre-enrichment ability of HQ. When the modification amount was further increased to 7 μL and 10 μL, the peak current decreased instead. This is because an overly thick hydrogel layer prolongs the electron transfer path, resulting in limited mass transfer. Therefore, 5 μL of OCZN-30 was selected as the optimal modification amount for the electrode, as this thickness best balanced electron transfer kinetics and diffusion behavior to achieve optimal sensor performance.

### 3.5 Mechanism analysis of HQ detection

As demonstrated in Figure 5A, the response of the OCZN-30/GCE to 100 μM HQ over the 5.5–8.5 pH range was evaluated using DPV. As the pH level increased, the peak potential underwent a shift towards a negative potential (Figure 5C). A linear relationship was observed between pH and potential, as depicted by the following equation: *E*_pa_ (V) = −0.055 *pH* + 0.43871 (R^2^ = 0.993). This finding suggests the involvement of protons in the redox process. The slope of the equation, measuring 55 mV/pH, approximates the Nernstian value of 59 mV/pH, indicating that the number of electrons and protons involved in the oxidation of HQ and protons are approximately equal. Furthermore, the DPV current value of HQ was observed to be at its maximum when the pH was 7.0 (Figure 5B). Subsequent analysis of these observations indicated that pH 7.0 was identified as the optimal condition.

An investigation was conducted into the effect of scanning rate on the catalytic reaction of HQ, with the range of the scanning rate investigated being 25–400 mV/s. As demonstrated in Figure 5D, the CV method was utilized to conduct this investigation. The following linear relationship is demonstrated between the redox currents (*I*_pa_ and *I*_pc_) and the square root of the scan rate (*v*^1/2^) (Figure 5E):


$$I_{\mathrm{pa}}\;(\mathrm{\mu A})=0.03263\;\nu^{1/2}+0.02409\;(R^{2}=0.996)$$

$$I_{\mathrm{pc}}\;(\mathrm{\mu A})=-0.5054-0.1080\;\nu^{1/2}\;(R^{2}=0.9900)$$

The relationship between *I*_pa_ and *I*_pc_ and *ν*^1/2^ is a primary function, thereby indicating that the action of HQ on the electrode is a diffusion-controlled process (42). Furthermore, it is demonstrated that *E*_pa_ and *E*_pc_ are linearly related to the logarithm of *ν* (ln*ν*). The following equations (Figure 5F):


$$E_{\mathrm{pa}}\;(V)=0.0349\;\ln\nu +0.3122\;(R^{2}=0.9910);$$

$$E_{\mathrm{pc}}\;(V)=-0.0255\;\ln\nu -0.1215\;(R^{2}=0.9963).$$

According to the Laviron equation (43), the electron transfer number *n* is calculated to be 1.82. The result of this experiment demonstrates that a total of two electrons are transferred during the redox of HQ. The electrochemical REDOX mechanism of HQ detected by OCZN-30/GCE is shown in Figure 5G, hydroquinone loses 2 protons and 2 electrons on the electrode surface and is converted into benzoquinone. These results confirm the high level of effectiveness of OCZN-30/GCE in reliably and rapidly detecting HQ.

### 3.6 Sensing performance of OCZN-30/GCE for HQ

Under optimized conditions, the electrochemical sensing of HQ was the subject of further evaluation by means of DPV technology. The performance of the prepared OCZN-30/GCE sensor in detecting HQ at different concentrations was evaluated by DPV. As demonstrated in the Figure 5H. The current response exhibited a proportional increase with the HQ concentration in 0.1 M PBS, within the range of 0.05–10 and 10–1800 μM. A linear calibration graph was obtained, showing a strong correlation between the oxidation peak current and the HQ concentration (Figure 5I). The regression equation is.


$$I_{\mathrm{pa}}\;(\mathrm{\mu A})=0.009412C\;(\mathrm{\mu M})+1.418\;(R^{2}=0.997)$$

$$I_{\mathrm{pa}}\;(\mathrm{\mu A})=0.0624\mathrm{6C}\;(\mathrm{\mu M})+1.0768\;(R^{2}=0.996)$$

The appearance of the two linear ranges reflects the transformation of the electrode surface control steps. In the low concentration range (0.05–10 μM), the adsorption sites on the surface of the OCZN-30/GCE electrode are far from saturation. HQ molecules are rapidly captured and enriched, and the electrochemical oxidation process is mainly controlled by adsorption, thus showing a high sensitivity. As the HQ concentration increases (10–1800 μM), the limited adsorption sites gradually become saturated. A large number of HQ molecules deposit on the electrode surface, leading to an increase in mass transfer resistance, and the diffusion process becomes dominant, thus the sensitivity decreases. The detection limit (LOD) of the developed sensor for HQ was calculated using the following formula: LOD is thus defined as 3 SD/k, where 3 represents the signal to noise ratio, SD indicates the standard deviation of the lowest concentration level of the HQ calibration curve, and k represents the slope of the standard curve. The calculated detection limit of the OCZN-30/GCE sensor for HQ is 0.0167 μM. Table 1 compares the performance of the OCZN-30/GCE sensor with various recently reported analytical methods in terms of HQ (44–49). Compared with most other reported methods for detecting HQ, the analytical performance of the constructed sensor is superior in terms of linear range or detection limit. The sensor’s outstanding detection performance stems from its unique material design and synergistic mechanism: ZIF-8-NH₂ acts as a crosslinker to form a stable three-dimensional heterogeneous interface with OSA/CMCS, and its porous structure and surface amino groups achieve efficient enrichment through physical adsorption and hydrogen bonding interactions with hydroquinone; The synergistic catalysis between Zn-N Lewis acid sites and surface amino groups markedly reduces the oxidation reaction energy barrier; the hydrophilicity of the hydrogel network facilitates mass transfer, enhancing substance exchange efficiency. These characteristics collectively enable highly sensitive and selective detection of hydroquinone.

**Table 1:** A comparison of analytical performance with different electrode materials for the determination of HQ.

Electrode materials	Linear range (μM)	Detection limit (μM)	Ref
TiO_2_/C_900_/GCEs	5–300	2.05	(44)
Sb-700/GCE	0.1–180	0.16	(45)
Co_3_O_4_@carbon/GCE	0.8–127.1	0.03	(46)
COFs/MWCNT/GCE	4–450	0.38	(47)
SSPC/GCE	5.0–25.0 25–1,200	0.18	(48)
VSe_2_@V_2_O_3_@V_2_CT_x_MXene	0.5–600	0.144	(49)
OCZN-30/GCE	0.05–10 10–1800	0.0167	This work

### 3.7 Reproducibility, repeatability, stability and anti-interference analysis

**Figure 6:**
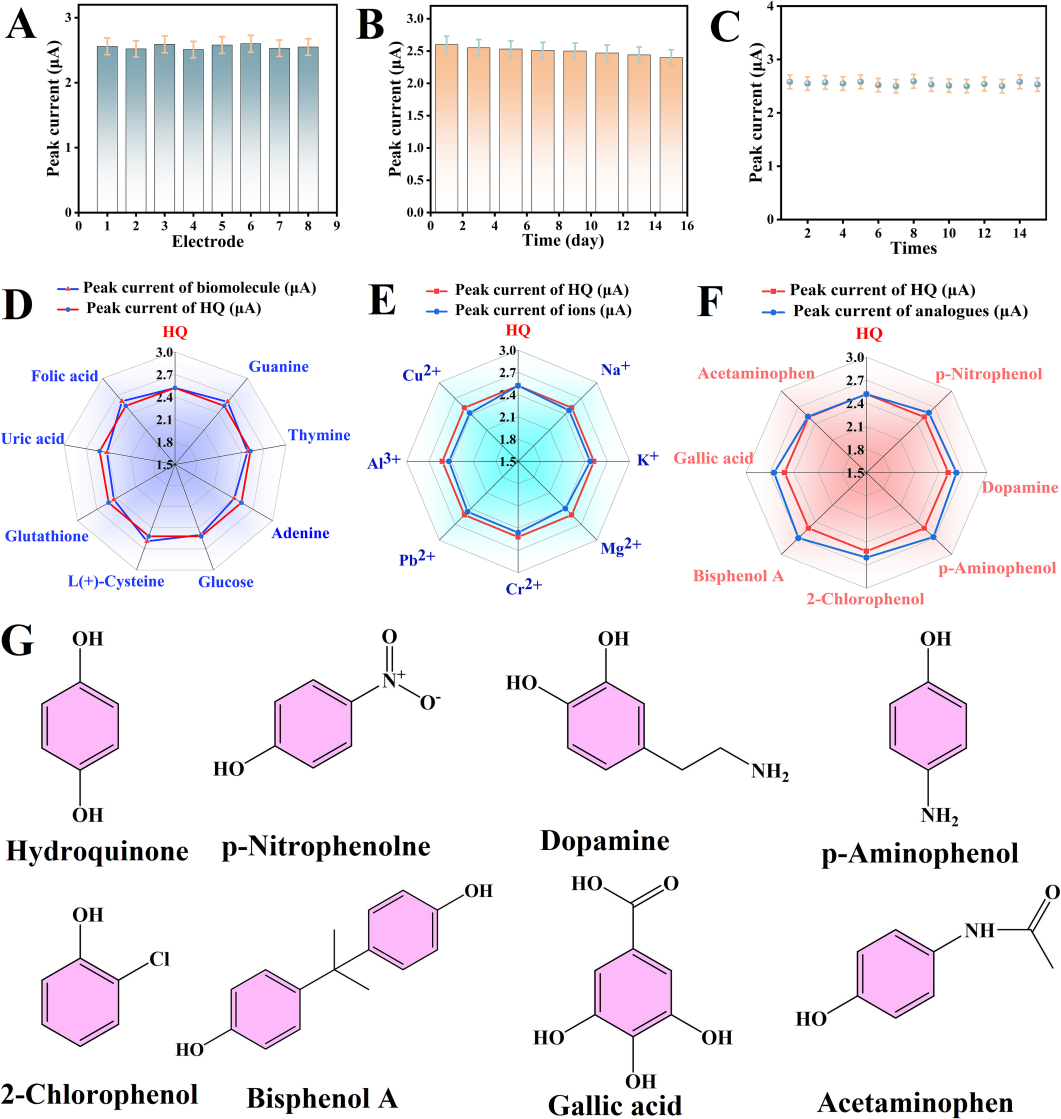
**(A)** Repeatability curves of different electrodes; **(B)** long-term stability test diagram of electrodes. **(C)** A current response diagram of 15 consecutive DPV tests of the electrodes, and **(D–F)** a radar diagram of the effects of different types of interferences on HQ. **(G)** Structural formula for structural analogs.

In addition, the long-term stability of the OCZN-30/GCE for HQ detection was the subject of further investigation. In Figure 6A, when eight individual OCZN-30/GCEs were prepared using the same methodology, it was determined, after cross-validation, that there was only a very small difference in the current response values reported by each electrode, with a relative standard deviation (R. S. D.) of the current values of 4.07%. With the current signal for HQ remaining above 90% after 15 days of maintenance in a controlled environment (Figure 6B). When a single OCZN-30/GCE was analyzed over 15 consecutive scans, the R.S.D. of the current response was calculated to be 3.89% (Figure 6C). The findings indicate that the developed sensor demonstrates excellent selectivity and stability.

As demonstrated in Figures 6D–F, the immunity of electrochemical sensors to interference is a pivotal parameter in determining their practical feasibility and reliability in selective analyte detection. The present study constitutes a systematic evaluation of the anti-interference performance of OCZN-30/GCE in the presence of several common interfering substances, as determined by the DPV method. Specifically, the following interferences have been identified:

other biomolecular interferences: guanine, thymine, adenine, glucose, L(+)-cysteine, glutathione, uric acid, and folic acid; ionic interferences: sodium (Na^+^), potassium (K^+^), magnesium (Mg^2+^), chromium (Cr^2+^), plumbum (Pb^2+^), aluminum (Al^3+^), and copper (Cu^2+^); and similar interferences: The following chemicals are of particular interest in this study: p-nitrophenol, dopamine, p-aminophenol, 2-chlorophenol, bisphenol A, gallic acid, and acetaminophen. The structural formulae of the analogues and biomolecules described are shown in Figure 6G. It is important to note that all interferents were added at an excess concentration of 50 times the target analyte concentration. In the presence of these potential interferents, the sensor demonstrated a high degree of stability in its response to HQ, with a maximum current deviation of ±3.08%. The findings suggest that the presence of these potential interferents does not significantly alter the electrochemical response of the sensor to HQ detection.

### 3.8 Actual sample analysis

It is crucial to study how the modified electrodes can be applied to real-time applications. We selected four types of water samples, namely tap water, drinking water, beverages and vegetable juice, for real sample analysis. The detection of HQ in water samples by DPV analysis. The unlabeled real samples did not show any oxidation peaks. After adding a known amount of HQ, we observed an increase in the oxidation current. The recovery rates of all real samples ranged from 98.0 to 104.0%, and the R. S. D. were all lower than 3.6% (Table 2). In addition, the accuracy of the sensor was confirmed by HPLC. Based on the HPLC results, the F-test was used to evaluate the homogeneity of variance to determine the differences between the two analytical methods. The t-test is also applied to investigate the error between the two means. The results of the F-test and t-test were both lower than the theoretical values, indicating that there was no significant difference between the results of the two methods. This proves that OCZN-30/GCE can accurately analyze actual samples.

**Table 2:** Detection of HQ in real samples (*n* = 3).

Samples	Added (μM)	Obtained by DPV^a^ (μM)	Recovery (%)	R.S.D.(%)	Obtained by HPLC^a^ (μM)	Calculated *F*-value^b^	Calculated *t*-value^c^
Tap water	0	Not found	–	–	–	–	–
	5	4.91 ± 0.048	98.0	2.3	4.91 ± 0.10	1.21	0.32
	15	15.35 ± 0.52	102.3	2.6	15.14 ± 0.25	1.61	0.37
	25	25.44 ± 0.37	101.8	1.7	25.39 ± 0.33	1.33	0.25
Drinking water	0	Not found	–	–	–	–	–
	5	4.87 ± 0.079	97.4	2.9	4.955 ± 0.17	1.21	0.12
	15	15.29 ± 0.45	101.9	2.0	15.55 ± 0.39	1.54	0.29
	25	25.89 ± 0.70	103.6	3.1	25.34 ± 0.41	1.30	0.18
Beverages	0	Not found	–	–	–	–	–
	5	5.14 ± 0.32	102.8	2.4	5.29 ± 0.30	1.15	0.22
	15	14.88 ± 0.25	99.2	2.7	14.40 ± 0.26	1.27	0.10
	25	25.72 ± 0.66	102.9	2.1	25.66 ± 0.43	1.38	0.41
Vegetable juice	0	Not found					
	5	4.93 ± 0.09	98.6	1.9	4.91 ± 0.15	2.65	1.42
	15	14.46 ± 0.31	96.4	2.5	14.50 ± 0.20	2.21	1.75
	25	25.55 ± 0.20	102.2	2.8	25.26 ± 0.32	1.53	1.88

^a^Three times independent evaluations. ^b^Confidence interval of F-value (95%, 19). ^c^Confidence interval of *t*-value (95%, 4.30).

## 4 Conclusion

In this study, a novel OCZN-30 electrochemical sensor based on a ternary composite hydrogel of OSA, CMCS and amino-functionalized ZIF-8 was successfully developed for the highly sensitive and selective detection of HQ in water samples. The outstanding performance of this sensor stems from the multi-level synergistic design of the material system. Covalent bonds resolve the challenges of dispersing and immobilizing MOF nanoparticles within the hydrogel matrix while establishing highly efficient electron transport pathways. Notably, the constructed OCZN-30 sensor exhibits outstanding linear response across the 0.05–1800 μM concentration range, with a detection limit as low as 16.7 nM (S/N = 3). It simultaneously demonstrates excellent specificity, repeatability, and reproducibility. Furthermore, satisfactory recovery rates were achieved when testing real samples via the standard addition method. In summary, the OCZN-30 sensor demonstrates significant potential for diagnostic analysis of HQ in water samples. This study not only expands the class of MOF-based composite hydrogels, but also demonstrates a broad application prospect in the field of food safety and security.

## Data Availability

The original contributions presented in the study are included in the article/supplementary material, further inquiries can be directed to the corresponding author/s.

## References

[1] Veerakumar P Sangili A Chen SM Kumar RS Arivalagan G Firdhouse MJ . Photocatalytic degradation of phenolic pollutants over palladium-tungsten trioxide nanocomposite. Chem Eng J. (2024) 489:151127. doi: 10.1016/j.cej.2024.151127

[2] Xie TY Liu QW Shi YR Liu QY. Simultaneous determination of positional isomers of benzenediols by capillary zone electrophoresis with square wave amperometric detection. J Chromatogr A. (2006) 1109:317–21. doi: 10.1016/j.chroma.2006.01.135, 16494888

[3] Pavel M Anastasescu C Atkinson I Papa F Balint I. Improved photocatalytic activity of Dion–Jacobson-type Tantalate perovskites modified with FeCl_2_. Materials. (2024) 17:4862. doi: 10.3390/ma17194862, 39410433 PMC11477869

[4] Guidi LR Tette PAS Fernandes C Silva LHM Gloria MBA. Advances on the chromatographic determination of amphenicols in food. Talanta. (2017) 162:324–38. doi: 10.1016/j.talanta.2016.09.068 27837837

[5] Cetinkaya F Yibar A Soyutemiz GE Okutan B Ozcan A Karaca MY. Determination of tetracycline residues in chicken meat by liquid chromatography-tandem mass spectrometry. Food Addit Contam Part B. (2012) 5:45–9. doi: 10.1080/19393210.2012.655782 24779694

[6] Robledo SN Pierini GD Nieto CHD Fernández H Zon MA. Development of an electrochemical method to determine phenolic monoterpenes in essential oils. Talanta. (2019) 196:362–9. doi: 10.1016/j.talanta.2018.12.069, 30683377

[7] Dhanjai Sinha A Kalambate PK Mugo SM Kamau P Chen J . Polymer hydrogel interfaces in electrochemical sensing strategies: a review. Trends Anal Chem. (2019) 118:488–501. doi: 10.1016/j.trac.2019.06.014

[8] Du HZ Dang XM Chen R Li YW Cui N Yang H. A universal three-dimensional hydrogel electrode for electrochemical detection of SARS-CoV-2 nucleocapsid protein and hydrogen peroxide. Biosens Bioelectron. (2024) 259:116355. doi: 10.1016/j.bios.2024.116355, 38754196

[9] Yang JT Wu DW Li JP Zhao CC Zhu L Xu CC . An injectable composite hydrogel of Verteporfin-bonded Carboxymethyl chitosan and oxidized sodium alginate facilitates Scarless full-thickness skin regeneration. Macromol Biosci. (2024) 24:e2300165. doi: 10.1002/mabi.202300165, 37681479

[10] Wang L Wang HN Dang HM Niu BL Yan H Guo RJ . An adhesive, antibacterial hydrogel wound dressing fabricated by dopamine-grafted oxidized sodium alginate and methacrylated carboxymethyl chitosan incorporated with cu(II) complex. Biomater Adv. (2025) 170:214217. doi: 10.1016/j.bioadv.2025.214217, 39929017

[11] Ma X Lin L Peng K Zheng Q Feng Y Chen Y. Construction and performance study of an injectable dual-network hydrogel dressing with inherent drainage function. ACS Appl Mater Interfaces. (2024) 16:59143–55. doi: 10.1021/acsami.4c09483, 39431566

[12] Qiu BW Cheng QQ Chen RK Liu CL Qin JC Jiang QX. Mussel-mimetic hydrogel coating with anticoagulant and Antiinflammatory properties on a poly(lactic acid) vascular stent. Biomacromolecules. (2024) 25:3098–111. doi: 10.1021/acs.biomac.4c00201, 38606583

[13] Zhang H Yuan W. Self-healable oxide sodium alginate/carboxymethyl chitosan nanocomposite hydrogel loading Cu^2+^-doped MOF for enhanced synergistic and precise cancer therapy. Int J Biol Macromol. (2024) 262:129996. doi: 10.1016/j.ijbiomac.2024.129996, 38342271

[14] Zhang J Wang Z. Nanoparticle–hydrogel based sensors: synthesis and applications. Catalysts. (2022) 12:1096. doi: 10.3390/catal12101096.

[15] Lu XY He SY Han ZN Guo X Pan XR Zhou YB. Metal-organic framework-based nanomaterials for biomedical applications. SmartMat. (2025) 6:e70055. doi: 10.1002/smm2.70055

[16] Chowdhury S Chattopadhyay MK Samanta P Banerjee P. The evolution of 2D metal-organic frameworks (2D MOFs): foundations and future prospects for next-generation lubricant additive design. ACS Appl Mater Interfaces. (2025) 17:62891–916. doi: 10.1021/acsami.5c14094, 41202206

[17] Sun W Liu JY Chu HC Wang Y. Controllable assembly of hollow interpenetrated zeolite imidazole framework nanocomposite for dopamine charge collection. Microchim Acta. (2023) 191:48. doi: 10.1007/s00604-023-06137-8 38141091

[18] Chong Y Ji LM Sun W Wang Y. Implantation of nano-MOFs into chitosan/sodium alginate hydrogels: boosting the electroanalytical response of chlorogenic acid in food samples. RSC Adv. (2025) 15:33592–600. doi: 10.1039/d5ra05652g, 40959301 PMC12434808

[19] Sun W Sun GR Liu JY Huang X Wang Y. Integration of MOF/COF core-shell composite material with wrinkled supramolecular hydrogel: a portable electrochemical sensing platform for noradrenaline bitartrate detection. Compos Part B Eng. (2025) 291:112029. doi: 10.1016/j.compositesb.2024.112029

[20] Zhang Z Li X Zhang S Zheng M Yuan F Li S . Simultaneous electrochemical and fluorescent in situ analysis of PD-1 on tumor cells via ZIF-8-enhanced quantum dot signal amplification. ACS Sens. (2025) 10:8829–38. doi: 10.1021/acssensors.5c02797, 41199544

[21] Ding R Zheng W Yang K Dai Y Ruan X Yan X . Amino-functional ZIF-8 nanocrystals by microemulsion based mixed linker strategy and the enhanced CO_2_/N_2_ separation. Sep Purif Technol. (2020) 236:116209. doi: 10.1016/j.seppur.2019.116209

[22] Zhao Q Sun Y Zhang J Fan F Li T He G . Mixed matrix membranes incorporating amino-functionalized ZIF-8-NH_2_ in a carboxylic polyimide for molecularly selective gas separation. J Membr Sci. (2024) 693:122326. doi: 10.1016/j.memsci.2023.122326

[23] Fan YL Liu HJ Wang ZL Wang ZL Zhu LL Wang YZ . A one-nano MOF-two-functions strategy toward self-healing, anti-inflammatory, and antibacterial hydrogels for infected wound repair. Chem Eng J. (2024) 497:155037. doi: 10.1016/j.cej.2024.155037

[24] Xu C Sun L Tong S Ouyang J Gu X. Cellulase immobilization on zeolitic imidazolate frameworks for boosting cellulose hydrolysis at high solids loading. Ind Crop Prod. (2023) 206:117693. doi: 10.1016/j.indcrop.2023.117693

[25] Wu Z He L Yan L Tan B Ma L He G . Hydrogels treat atopic dermatitis by transporting marine-derived miR-100-5p-abundant extracellular vesicles. ACS Biomater Sci Eng. (2024) 10:7667–82. doi: 10.1021/acsbiomaterials.4c01649 39585960

[26] Xiong Y Deng N Wu X Zhang Q Liu S Sun G. De novo synthesis of amino-functionalized ZIF-8 nanoparticles: enhanced interfacial compatibility and pervaporation performance in mixed matrix membranes applying for ethanol dehydration. Sep Purif Technol. (2022) 285:120321. doi: 10.1016/j.seppur.2021.120321

[27] Gong H Tang L Chen C Chen F Cai C. Portable paper-based molecularly imprinted sensor for visual real-time detection of influenza virus H_5_N_1_. Chem Eng J. (2023) 477:146990. doi: 10.1016/j.cej.2023.146990

[28] Liu X Hu J Hu Y Liu Y Wei Y Huang D. Multifunctional injectable oxidized sodium alginate/carboxymethyl chitosan hydrogel for rapid hemostasis. Colloids Surf B Biointerfaces. (2025) 245:114346. doi: 10.1016/j.colsurfb.2024.114346, 39486372

[29] Fan LH Pan XR Zhou Y Chen LY Xie WG Long ZH . Preparation and characterization of crosslinked carboxymethyl chitosan–oxidized sodium alginate hydrogels. J Appl Polym Sci. (2011) 122:2331–7. doi: 10.1002/app.34041

[30] Park J Nam J Yun H Jin HJ Kwak HW. Aquatic polymer-based edible films of fish gelatin crosslinked with alginate dialdehyde having enhanced physicochemical properties. Carbohydr Polym. (2021) 254:117317. doi: 10.1016/j.carbpol.2020.117317 33357880

[31] Zhao L Feng Z Lyu Y Yang J Lin L Bai H . Electroactive injectable hydrogel based on oxidized sodium alginate and carboxymethyl chitosan for wound healing. Int J Biol Macromol. (2023) 230:123231. doi: 10.1016/j.ijbiomac.2023.123231, 36641017

[32] Zhao Q Niu K Wang RN Lian SH Li R Zang GL . Pre-anchoring matrix onto zeolitic imidazolate frameworks towards defect-free mixed matrix membranes for efficient CO_2_ separation. J Membr Sci. (2023) 683:121869. doi: 10.1016/j.memsci.2023.121869

[33] Liu JY Sun W Sun GR Huang X Lu S Wang Y. Portable electroanalytical platform based on eco-friendly biomass-based hydrogels with bimetallic MOF composites for trace acetaminophen determination. ACS Biomater Sci Eng. (2024) 11:649–60. doi: 10.1021/acsbiomaterials.4c01751, 39694669

[34] Liu JY Sun GR Sun W Zha XQ Wang N Wang Y. Portable electrochemical sensor for adrenaline detection using CoNi-MOF-based CS-PAM hydrogel. J Colloid Interface Sci. (2024) 671:423–33. doi: 10.1016/j.jcis.2024.05.195, 38815377

[35] Huang X Wang N Liu J Sun W Sun GR Zhang Z . Flexible hydrogels with in-situ grown cobalt-based metal-organic frameworks for high-performance electrochemical detection of 2,4,6-trichloropshenol. Chin Chem Lett. (2025) 37:112067. doi: 10.1016/j.cclet.2025.112067

[36] Zheng DY Zhang CY Chen ZW Zhu PZ Gao CX. Tough and anti-swelling γ-polyglutamic acid/polyvinyl alcohol hydrogels for wearable sensors. J Appl Polym Sci. (2023) 140:e53792. doi: 10.1002/app.53792

[37] Biswas S Chen Y Xie Y Sun X Wang Y. Ultrasmall au(0) inserted hollow PCN-222 MOF for the high-sensitive detection of estradiol. Anal Chem. (2020) 92:4566–72. doi: 10.1021/acs.analchem.9b05841, 32077686

[38] Sun GR Sun W Liu JY Zha XQ Lu S Wang Y. Chitosan-based hydrogel functionalized with Fe(II) phthalocyanine for butylated hydroxyanisole determination. Inorg Chem. (2024) 63:17263–73. doi: 10.1021/acs.inorgchem.4c03086, 39222464

[39] Natarajan S Manna K. Bifunctional MOFs in heterogeneous catalysis. ACS Org Inorg Au. (2024) 4:59–90. doi: 10.1021/acsorginorgau.3c00033, 38344010 PMC10853920

[40] Chen YL Sun X Biswas S Xie Y Wang Y Hu X. Integrating polythiophene derivates to PCN-222(Fe) for electrocatalytic sensing of L-dopa. Biosens Bioelectron. (2019) 141:111470. doi: 10.1016/j.bios.2019.111470, 31252260

[41] Gonzalez-Sanchez MI Rubio-Retama J Lopez-Cabarcos E Valero E. Development of an acetaminophen amperometric biosensor based on peroxidase entrapped in polyacrylamide microgels. Biosens Bioelectron. (2010) 25:164–70. doi: 10.1016/j.bios.2010.03.024 20382517

[42] Chen YL Huang W Chen K Zhang T Wang Y Wang J. A novel electrochemical sensor based on core-shell-structured metal-organic frameworks: the outstanding analytical performance towards chlorogenic acid. Talanta. (2019) 196:85–91. doi: 10.1016/j.talanta.2018.12.033, 30683415

[43] Li YG Zhang L Dang YY Chen ZQ Zhang RY Li YC . A robust electrochemical sensing of molecularly imprinted polymer prepared by using bifunctional monomer and its application in detection of cypermethrin. Biosens Bioelectron. (2019) 127:207–14. doi: 10.1016/j.bios.2018.12.002, 30611108

[44] Wang Z Li M Ye Y Yang Y Lu Y Ma X . MOF-derived binary mixed carbon/metal oxide porous materials for constructing simultaneous determination of hydroquinone and catechol sensor. J Solid State Electrochem. (2018) 23:81–9. doi: 10.1007/s10008-018-4111-z

[45] Jiang X Yuan Y Zhao X Wan C Duan Y Wu C. Microbial synthesis of antimony sulfide to prepare catechol and hydroquinone electrochemical sensor by pyrolysis and carbonization. Environ Res. (2024) 252:1188. doi: 10.1016/j.envres.2024.1188 38582422

[46] Khan M Ahmad S Kamal T Sameer S Khan SB. Development of a Co_3_O_4_: SnO_2_ nanocomposite-modified GCE for sensitive and stable electrochemical detection of organophosphate pesticides. Environ Technol Innov. (2025) 38:104081. doi: 10.1016/j.eti.2025.104081

[47] Liu B Guo H Sun L Pan Z Peng L Wang M . Electrochemical sensor based on covalent organic frameworks/MWCNT for simultaneous detection of catechol and hydroquinone. Colloids Surf A Physicochem Eng Asp. (2022) 639:128335. doi: 10.1016/j.colsurfa.2022.128335

[48] Liu Y Zhang ZJ Li YS Shi F Ai YJ Wang BL . Electrochemical detection of hydroquinone based on marine biomass carbon from shrimp shells as electrode modifier. Int J Electrochem Sci. (2023) 18:100063. doi: 10.1016/j.ijoes.2023.100063

[49] Han Y Zhang L Li S Li F Xin J Wang X . Controllable selenization strategy for in-situ construction of V_2_CT_x_ MXene-based multiphase electrode materials for simultaneous detection of hydroquinone and catechol in drinking water and beverages. Food Chem. (2025) 483:144234. doi: 10.1016/j.foodchem.2025.14423 40215744

